# The Threat of Food Additive Occurrence in the Environment—A Case Study on the Example of Swimming Pools

**DOI:** 10.3390/foods12061188

**Published:** 2023-03-11

**Authors:** Anna Lempart-Rapacewicz, Edyta Kudlek, Katarzyna Brukało, Rafał Rapacewicz, Łukasz Lempart, Mariusz Dudziak

**Affiliations:** 1 Department of Water and Wastewater Engineering, Faculty of Energy and Environmental Engineering, Silesian University of Technology, Konarskiego 18, 44-100 Gliwice, Poland; 2 Department of Health Policy, School of Health Sciences in Bytom, Medical University of Silesia in Katowice, Piekarska Street 18, 41-902 Bytom, Poland; 3 Underwater Activities Center Association “Nurek Bytom”, Chorzowska 28a, 41-902 Bytom, Poland

**Keywords:** food additives, organic micropollutants, swimming pools, ecotoxicity, health risk

## Abstract

Widespread use and the continuous increase in consumption has intensified the presence of food additives and their metabolites in the environment. The growing awareness that newly identified compounds in the environment may cause a real threat, both to the environment and to future generations due to the transformation they undergo in ecosystems, makes this topic a leading problem of engineering and environmental protection. This manuscript highlights the relevance of finding these compounds in water. The exposure routes and the threat, both to human health and to the aquatic environment, have been discussed. The research presented in the article was aimed at determining the degree of contamination of swimming pools with food additives. Thirteen food additives have been identified in ten tested pools. The most frequently found were antioxidants (E320, E321) and preservatives (E211, E210), which were present in all of the tested swimming pools, both public and in private backyards. Ascorbic acid (E300) and citric acid (E330) occurred in all of the tested private swimming pools, while aspartame (E951, sweetener) and canthaxanthin (E161g, colour) were identified only in private pools. The hazard statements according to the European Chemicals Agency indicate that the identified compounds may cause both immediate effects (skin or eye irritation, allergic reactions) and also long-lasting effects, e.g., damaged fertility or genetic defects.

## 1. Introduction

Food additives are substances that are not normally consumed as food itself but are added to food intentionally for a technological purposes. According to EU Regulations on food additives [1], they must be safe when used. Most of them are only permitted to be used in certain foods and are subject to specific quantitative limits in conjunction with the appropriate legislation. Among those approved in the EU, the Food Standards Agency distinguishes substances belonging to groups of compounds, such as colours, preservatives, antioxidants, sweeteners, emulsifiers, stabilizers thickeners and other types. These kinds of substances only partially undergo a biotransformation processes in particular phases of metabolism in the human body [2]. Therefore, they get into the sewage and sewage treatment plants in unchanged form, or their metabolites do. In the meantime, under the influence of many different factors, they undergo numerous transformations and chemical reactions, such as oxidation or photodegradation processes. Traditional sewage treatment systems are not able to retain most of these contaminants and their by-products, which results in the accumulation of these organic micropollutants in water, soil, air (in the case of volatile substances), plants and animal bodies. The continuous increase in consumption has intensified the presence of food additives and their metabolites in the environment. As a result, the latest literature [3,4] classifies food additives as one of the groups of so-called Contaminants of Emerging Concern (CECs), defined by the United States Environmental Protection Agency (USEPA) and United States Geological Survey (USGS) as any chemical substance detected in particular elements of the environment and previously not naturally occurring in them [5,6]. The development of analytical techniques allows for the separation of chemicals with very low concentrations from environmental samples with increasing efficiency, causing a growing awareness that newly identified compounds in the environment classified as CECs may cause a real threat, both to the environment and to future generations. The exposure of organisms to environmental factors (including organic pollution) is the main cause of numerous dysfunctions, diseases and premature death. This applies to both plant and animal organisms, as well as the human population. This makes CECs a leading problem of engineering and environmental protection. This group of contaminants cover such a broad classification framework that it is impossible to clearly indicate the number of compounds currently included in this group. Meijer et al. [7]. developed counting software that identified, in total, 69,526 compounds with a CAS number and 306,279 different metabolites of them occurring in the environment.

**Figure 1:**
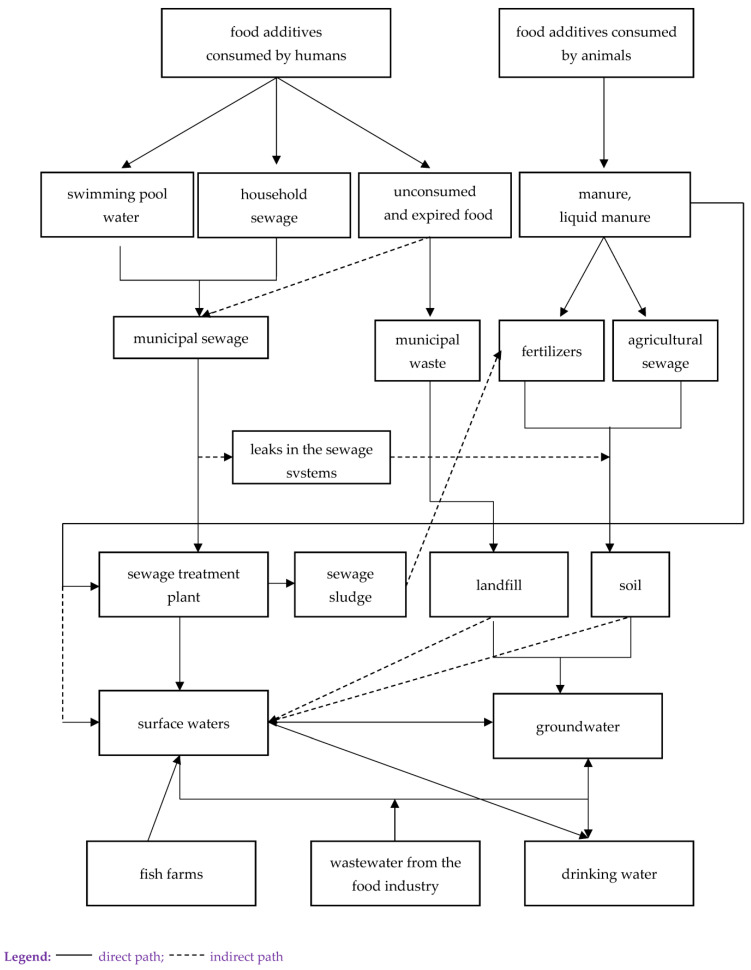
Pathways of food additive penetration into the environment.

Migration paths of CECs (including food additives) into the environment are very different. Generally, their sources are divided into point and non-point types [8]; the main and most common of them are municipal, agricultural and industrial sewage, as well as leachate from landfills and the food industry. One possible means of food additives getting into the environment as its pollutant are shown in Figure 1.

Significant loads of food additives introduced into the environment in an uncontrolled way are carried by sewage from swimming pools. This applies to both the discharge of water from swimming pool basins and the discharge of washings from the backwashing of filters [9,10,11]. These are very special types of water streams because a number of different anthropogenic pollutants introduced by swimmers then get into the pool water, including not only food additives, but also Pharmaceuticals and Personal Care Products (PPCPs) [12,13], industrial additives and flame retardants [14,15]. Due to the specificity of the operation of swimming pool installations and treatment plants, in this particular water environment, pollutant accumulation processes, many physical transformations and chemical reactions occur (i.e., oxidation, chlorination, photodegradation), leading to the formation of very harmful by-products [16], including disinfection by-products (DBP), for example trichloromethane or pentachlorophenol, that are not only classified as priority substances in the field of water policy [17] particularly harmful to the environment but may also pose a serious threat to human health [18,19,20] due to their genotoxic and carcinogenic properties. Nowadays, the literature already describes over 600 harmful DBPs formed in swimming pools. These include: haloacetic acids (mostly di- and trichloroacetic acids), haloacetonitriles (dichloroacetonitrile, dibromoacetonitrile, trichloroacetonitrile), halobenzoquinones, halonitromethanes, N-nitrosamines, as well as chloral hydrate and bromine hydrate, cyanide halides and chloropicrin. In addition, when inorganic bromides are present in pool water (e.g., seawater pools or brines), they may oxidize and participate in the reaction, leading to the formation of brominated by-products. Food additives due to their chemical structure, if present in swimming pool water, may have the potential to form these types of potentially harmful compounds.

The research presented in this paper was aimed at determining the degree of contamination of swimming pools with food additives and their transformation products. In addition, an assessment of the threat caused to the environment by this phenomenon and the human exposure routes have been discussed. Determination of the extent of living organisms’ exposure to chemical contaminants is crucial to the evaluation of their adverse effects on both the environment and human health.

## 2. Materials and Methods

Samples of swimming pool water collected from 10 different outdoor swimming pools were subjected to broad-spectrum, non-target chromatographic analysis (NTCA), the aim of which was to identify as many compounds as possible by comparing the obtained mass spectra with reference spectra collected in the commercial NIST v.17 database. Food additives were selected from the identified chemical compounds, and then swimming pool water samples were subjected to qualitative targeted chromatographic analysis (TCA), during which their presence in the tested pools was confirmed by the injection of every identified compound’s analytical reference standard. For compounds with confirmed occurrence in the tested pools, a quantitative analysis was carried out, determining their concentration levels in swimming pool water.

Swimming pool fill water (fresh water) was also collected to assess the source of swimming pool water contamination.

### 2.1. Materials and Equipment

Chromatographic analyses were carried out using Gas Chromatograph with Mass Spectrometry detector (GC-MS) by Agilent Technologies (Santa Clara, CA, USA) equipped with capillary columns by Sigma-Aldrich (Poznań, Poland).

Disposable Superclean^TM^ extraction tubes by Merck KGaA (Darmstadt, Germany) and organic solvents methanol (MeOH), acetonitrile (ACN) and dichloromethane (DCM) with a purity over 99% from Avantor Performance Materials Poland S.A. (Gliwice, Poland) were used for Solid Phase Extraction (SPE).

The deionized water was obtained from a laboratory water distillation station Arium Comfort II UV by Sartorius AG (Göttingen, Germany).

The analytical reference standards of food additives used during targeted chromatographic analysis (TCA) were delivered by Merck KGaA (Darmstadt, Germany).

The obtained mass spectra were compared with the United States National Institute of Standards and Technology NIST v17 Mass Spectral Library using MassHunter software.

### 2.2. Research Objective

Environmental samples of swimming pool water were taken in accordance with the guidelines of the PN-EN ISO 5667-3:2018-08 standard [21], from outdoor swimming pools (five public and five private backyards facilities, SP1–SP10). The characteristic parameters of the sampled swimming pools are listed in Table 1. Water samples were collected in dark glass bottles, secured for transport in accordance with the procedures [21] and immediately transported to the Center of New Technologies of the Silesian University of Technology, where they were immediately prepared for analysis.

**Figure 2:**
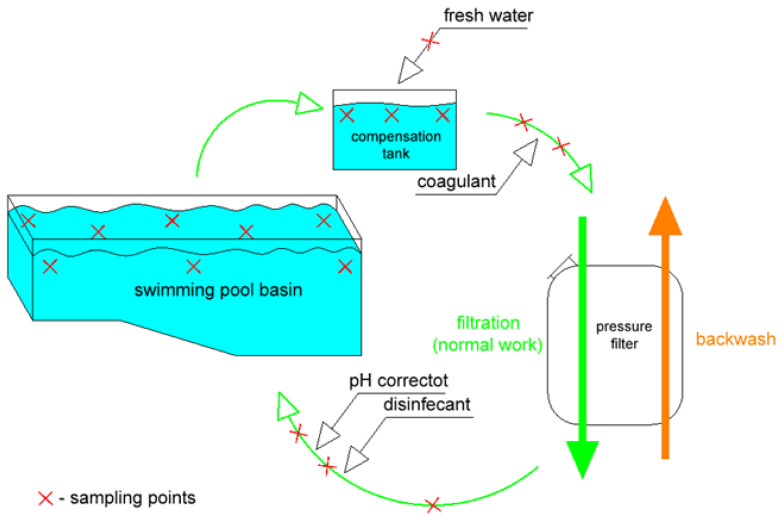
Sampling points of water for research.

Due to the experience from earlier authors’ studies [22,23] and the dependence of chemical concentrations on the sampling point location in swimming pool basins [12,23], samples were taken from various characteristic points of both the basin, the installation and the pool water treatment system (Figure 2), and an average mixed sample was used for the analyses. The sampling points were selected in such a way as to ensure the reliability of the reported results, as during the processes taking place in the swimming pool water system, changes of micropollutants may occur.

Swimming pool fill water (fresh water) samples were analysed separately.

The pools tested in the presented study were selected in such a way that the water treatment procedures (disinfection, coagulation, filtration and pH adjustment) were comparable in all of them. This means that sodium hypochlorite was used at the disinfection stage, no methods of disinfection support were used, the same type of coagulant and pH corrector was used, and filtration was carried out in pressure filters filled with a classic sand bed.

### 2.3. Chromatographic Determination of Food Additives in Swimming Pool Water

The collected swimming pool water samples were subjected to chromatographic analysis using GC-MS, which was preceded by solid phase extraction (SPE) according to the authors’ own procedure [24] that allows for the extraction of the most possible analytes present in the sample.

Detailed parameters of the extraction processes are presented in Table 2 and conditions of GC-MS analysis are presented in Table 3.

### 2.4. The Decomposition Processes for Toxicity Change Evaluation

The laboratory experiments were conducted in model conditions in order to show the changes in the toxicity of aqueous solutions of food additives during the decomposition processes taking place in swimming pool water installations. In swimming pool water, the toxicity effect is always 100% for each sample due to the presence of free chlorine. This is why the toxicity tests for real conditions are not applicable.

For this purpose, a laboratory swimming pool water treatment system was used, equivalent to the scheme of the tested pools (Figure 2), including coagulation, filtration, disinfection and pH adjustment. The sodium thiosulfate was used to stop the chlorination process and to avoid the influence of the presence of free chlorine on the toxicity of the samples. 

Toxicity effect have been measured by the use of Microtox^®^ bioassay based on the measurement of the changes in the intensity of light emission by selected strains of the bioluminescent bacteria Aliivibrio fischeri. These bacteria are widely used bioindicators due to their high sensitivity for a broad range of toxicants, including different groups of organic micropollutants. Details of the procedure are given in [15]. The test was conducted according to the Screening Test procedure, which allow for the estimation of the toxic effect of tested water samples comparative to a reference nontoxic sample. The reference sample was a 2% NaCl solution. The obtained test results of the toxicological effect of the compound water solutions allowed us to classify them into particular toxicity classes. 

## 3. Results

As a result of qualitative NTCA, several hundred different mass spectra were obtained, the comparison of which, with reference spectra collected in the commercial database NIST v.17, allowed the identification of over 100 different organic micropollutants with a probability of a correct match of over 70%. They included not only food additives but also pharmaceuticals, personal care products, industrial additives and others. In addition, a group of micropollutants, which seem to be intermediate or final products of transformations or processes taking place in swimming pool installations, was separately identified. 

The list of selected compounds from the group of food additives identified in the analysed samples with a match probability above 70% is presented in the Table 4. They have been classified according to the classification given by the Food Standards Agency [25] as colours, preservatives, antioxidants, sweeteners and others. No compounds from the group of emulsifiers, stabilizers, thickeners and gelling agents have been identified in this study.

No food additives were detected in any of the fill water (fresh water) samples.

**Figure 3:**
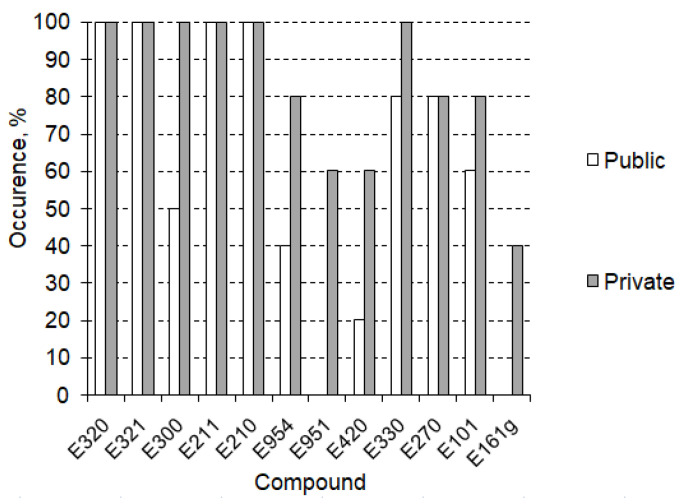
Food additives occurrence in public and private backyard swimming pools (N = 10).

**Figure 4:**
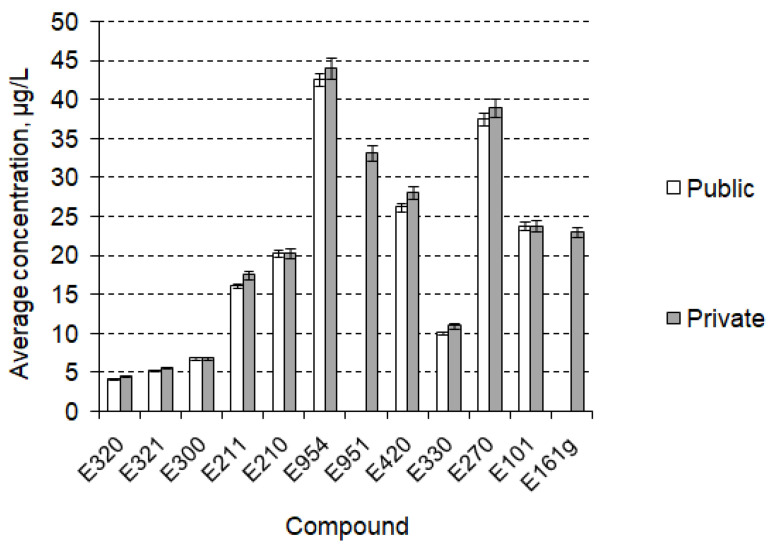
Concentration levels of food additives identified in tested swimming pools.

The further stage of research, including qualitative TCA, confirmed the presence of the listed compounds in the tested pools (Figure 3) and allowed the determination of their concentration levels, as presented in the Figure 4. Concentration levels are expressed a mean values calculated as the arithmetic mean of the measured concentrations in the pools where the compound was identified. Each concentration had been measured as the average of three consecutive concentration measurement replicates. The measurement errors marked on the chart are the standard deviation of the repetitions made. Error values did not exceed 5%.

The values of the validation parameters for the determination of the concentrations of the tested compounds are summarized in Table 5. The Instrumental Detection Limit (IDL) for the tested compounds was determined on the basis of the quotient of the signal from a given compound recorded by the chromatograph detector and the noise from the coastline (Signal to Noise Ratio, SNR) equal to 3. The Recovery (R) was determined using the optimal extraction procedure for each test compound. The validation of the method was carried out for various concentrations of compounds, enabling the determination of the Coefficient of Variation (CV, value in the range of 1–3% confirms the high repeatability of the method) and the Limit of Quantification (LOQ), for which the SNR was assumed equal to 10.

## 4. Discussion

Food additives were found to be above the LOQs in all of the tested swimming pools, while they all were below the LOQs in all of the fill water samples. This implies that contamination of swimming pool water by food ingredients is occurring within the swimming pools themselves and is likely due to human-derived sources, such as through swimmers’ excretion of body fluids (accidental urinary excretion or sweat) or accidentally putting food in the water (e.g., falling into the swimming pool).

In general, food additives occurred more often in private swimming pools than in public ones. This results from the different specificity of the use of these two types of facilities, a lesser sanitary regime in private pools and more advanced methods of water treatment in public ones. The most frequently identified compounds in the studied basins were antioxidants (E320, E321) and preservatives (E211, E210); they were present in all of the tested swimming pools, in both public and private backyards, while the antioxidants’ average concentration levels were the lowest of all tested contaminants (respectively, 4.1 ± 0.3 µg/L and 5.2 ± 0.2 µg/L). Ascorbic acid (E300) and citric acid (E330) occurred in all of the studied private swimming pools and over half of public ones (3 of 5 and 4 of 5, respectively), while aspartame (E951, sweetener) and canthaxanthin (E161g, colour) were identified only in private pools (respectively, 3 of 5 and 2 of 5). The highest concentration level (42.6 ± 0.4 µg/L) was measured for saccharin (E954), which was present in 60% of tested swimming pools (40% of publics and 80% of privates). Sorbitol occurred in 40% of tested pools (1 of 5 public and 4 of 5 private) with average concentrations equal to 26.2 ± 0.3 µg/L. Lactic Acid was observed in 80% of facilities (4 of 5 of both public and private) at a concentration level of 37.5 ± 0.4 µg/L and Riboflavin in 70% (3 of 5 public and 4 of 5 private) with a concentration of 23.8 ± 0.2 µg/L.

The levels of the measured and presented concentrations in this paper are likely affected by many factors, including the function of the pool basin, water temperature, the number and demography of users, types of activities carried out, exposure of basin to sunlight and the type of disinfection used (such as the incorporation of UV disinfection) or the type of tested compound. This was similarly observed in studies on the presence of micropollutants from other groups in swimming pool water [26,27,28]. Variation of food additive concentrations during the day was also observed, described by Teo et al. on the example of caffeine [29].

All of the detected compounds are approved for use in food, which means they are relatively safe for human health. However, for a number of reasons, their presence in the environment is of concern. The phenomenon of the co-occurrence of compounds from different groups simultaneously in tested pool basins is alarming. Some studies were focused on the potential interactions and synergies of food additives [30]. For example, McCannet et al. [31] pointed out that a mixture of sodium benzoate with colourings can cause increased hyperactivity in children.

Hazard statements of the food additives identified in the tested swimming pools, according to the Globally Harmonized System of Classification and Labelling of Chemicals (GHS), based on European Chemicals Agency (ECHA) data, are presented in Table 6. The collected data show that the tested compounds may affect different parts of the human body and also the aquatic organism. Human exposure via different routes may cause not only immediate effects, such as skin or eye irritation and allergic reactions, but also long-lasting effects, e.g., damaged fertility or genetic defects. The available literature data highlight three main ways of exposing swimmers to organic compounds (including food additives) and their by-products present in swimming pool water:

- Oral route—by the direct swallowing of water. Studies presented in [32] showed that during 45 min of swimming, an average adult swallows 16 mL of pool water, while an average child swallows 37 mL;
- Inhalation route—by inhalation of volatile or aerosolized substances dissolved. Among them, volatile by-products, which are formed as a result of the reaction of organic compounds (including food additives) with chlorine compounds. It should be noted that, due to the properties of these compounds and substances, they accumulate in swimming pools just above the water table, in the so-called swimmer’s breathing zone (exactly where the swimmer takes a breath). Research has proven the cause of respiratory illness (e.g., asthma) among professional swimmers. Literature reports indicate that even a short exposure to some of the chlorinated by-products causes coughing or severe irritation of the respiratory tract of swimmers. It can also cause changes in biomarkers in the lungs [33];
- Dermal route—by direct contact or skin absorption. Some contaminants occurring in swimming pool water, or their by-products, can directly affect the skin, eyes or mucous membranes, and some can also penetrate the skin and be absorbed by the body. For example, E321 does penetrate the skin [34]. The extent of such absorption depends on a number of factors, including the time of contact with water, its temperature or the concentration of the absorbed chemical substance.

**Table 6:** Health hazards classification of food additives identified in tested swimming pools [35].

Abbreviation	Hazard Statement Code	Hazard Class	Category
E320	H302	Harmful if swallowed	4
	H315	Causes skin irritation	2
	H317	May cause an allergic skin reaction	1
	H319	Causes serious eye irritation	2
	H335	May cause respiratory irritation	3
	H351	Suspected of causing cancer	2
	H361	Suspected of damaging fertility or the unborn child	2
	H400	Very toxic to aquatic life	2
	H411	Toxic to aquatic life with long lasting effects	1
E321	H302	Harmful if swallowed	4
	H312	Harmful in contact with skin	4
	H315	Causes skin irritation	2
	H317	May cause an allergic skin reaction	1
	H319	Causes serious eye irritation	2
	H335	May cause respiratory irritation	3
	H340	May cause genetic defects	1B
	H351	Suspected of causing cancer	2
	H361	Suspected of damaging fertility or the unborn child	2
	H370	Causes damage to organs	1
	H373	May cause damage to organs through prolonged or repeated exposure	2
	H400	Very toxic to aquatic life	1
	H410	Very toxic to aquatic life with long lasting effects	1
	H413	May cause long lasting harmful effects to aquatic life	4
E319	H302	Harmful if swallowed	4
	H312	Harmful in contact with skin	4
	H315	Causes skin irritation	2
	H317	May cause an allergic skin reaction	1
	H319	Causes serious eye irritation	2
	H335	May cause respiratory irritation	3
	H400	Very toxic to aquatic life	1
	H410	Very toxic to aquatic life with long lasting effects	1
E300	H314	Causes severe skin burns and eye damage	1
	H315	Causes skin irritation	2
	H319	Causes serious eye irritation	2
	H318	Causes serious eye damage	1
E211	H319	Causes serious eye irritation	1
E210	H302	Harmful if swallowed	4
	H315	Causes skin irritation	2
	H318	Causes serious eye damage	1
	H319	Causes serious eye irritation	2
	H372	Causes damage to organs (lungs) through prolonged or repeated exposure by inhalation	1
E954	H315	Causes skin irritation	2
	H317	Causes serious eye damage	1
	H318	May cause an allergic skin reaction	1
	H341	Suspected of causing genetic defects	2
	H351	Suspected of causing cancer	2
	H361	Suspected of damaging fertility or the unborn child	2
E951	H312	Harmful in contact with ski	4
	H332	Harmful if inhaled	4
	H372	Causes damage to organs through prolonged or repeated exposure	1
E420	Not Classified	-	-
E101	H302	Harmful if swallowed.	0
E161g	Not Classified	-	-
E330	H302	Acute Toxicity	4
	H315	Skin corrosion/irritation	2
	H318	Eye damage/eye irritation	1
	H335	May cause respiratory irritation	3
E270	H315	Skin corrosion/irritation	1
	H318	Eye damage/eye irritation	1

Swimming pool water is only one of many sources of environmental pollution with organic compounds classified as food additives and their by-products. Pollutants entering ecosystems in trace concentrations through various routes (including effluent from swimming pool facilities) accumulate in the environment, causing a continuous increase in environmental concentrations, as well as an increase in the exposure of plant, animal and human organisms.

Based on the methodology proposed by Fantuzzi et al. [36], for the determined concentration levels of the tested organic micropollutants, the human health risk assessment was carried out, for children (3 years), teenagers (14 years) and adults, dividing each age group by gender. The average body weight for the individual analysed groups was determined according to Cacciari et al. [37], taking into account the 50th percentile. The average volume of water swallowed by swimmers was adopted according to Dufour et al. [32]. The worst-case scenario was predicted assuming the maximum measured concentration of each micropollutants tested and assuming daily use of the pool by users. The hazard factors for swimmers of all ages and genders were less than 0.001, indicating that the health risk from oral exposure to the tested compounds in swimming pools is low, considering exposure to a single contamination with individual compounds. However, it should be highlighted and kept in mind that in swimming pool basins there is co-exposure to countless amount of different organic micropollutants, which is not taken into account in this health risk assessment methodology. It must also be taken into account that some of these food additives bioaccumulate, so with frequent use of the pool, the actual health risk may be much higher.

Table 7 provides single chemical environmental toxicity data of the identified compounds on aquatic species collected by the use of ECOTOX Knowledgebase of the United States Environmental Protection Agency EPA [38].

Butylated hydroxyanisole (E320), butylated hydroxytoluene (E321) and tertiary butylhydroquinone (E319) are the most widely used food antioxidants due to their low cost, high performance, and wide availability. They can be found in a great variety of products, e.g., oils, margarines and fat-containing products. They are approved food antioxidants in the European Union, the United States, Australia, New Zealand and many other regions [39,40]. Previous studies show that these antioxidants and their transformation products have been found in a concentration level of 10÷2000 ng/L in the water environment (rivers, ocean, ground water and wastewater, sewage, sludge, sediment). They have also been detected in indoor dust, sludge, sediments, molluscs and human plasma and nails [39,41,42,43,44,45,46]. Human exposure to synthetic phenolic antioxidants (including E319, E320 and E321) is of high interest due to their reported toxicity effects. For example, E320 can disrupt the endocrine system [47,48] and according to the release in2021 of the Fifteenth Edition of the Report on Carcinogens by the U.S. Department of Health and Human Services, it is reasonably anticipated to be a human carcinogen based on sufficient evidence of carcinogenicity from studies in experimental animals [49]. It has also been found that E321 interferes with satiety signals sent from the digestive system to the brain, which may cause individuals to eat more than they otherwise would, potentially leading to obesity [43]. In addition, both E320 and E321 can easily be transformed to tert-butylhydroquinone (E319) by oxidation reaction [39] and further degrade due to irradiation. Accumulation ofE319 in body tissues is negligible; however, it is noteworthy that it possibly leads to nutritional disorders and chronic diseases and adverse biological effects on human health at high doses or in the long-term. E319 can have side effects on human health through activation of inflammatory routes, generation of reactive species, induction of CYP1A1, activation of caspases and decreases in GSH/ATP levels, and triggering of the gradual development of cancers [50]. 

**Figure 5:**
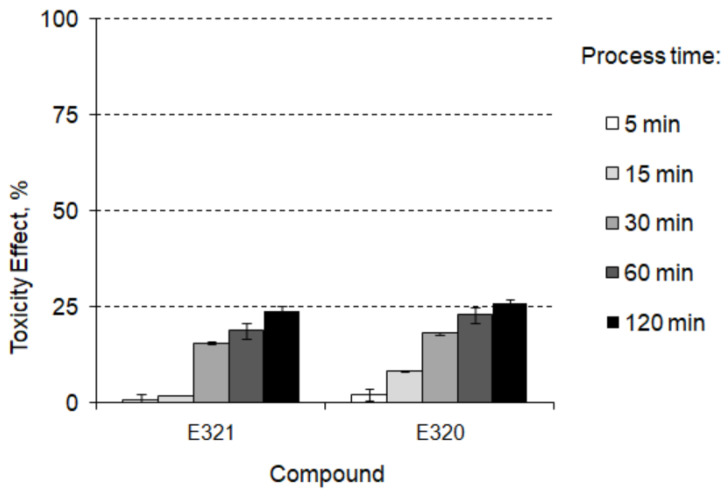
The toxicity change in the by-product formation pathway in swimming pool water installation.

The laboratory experiment conducted in model conditions showed the increase in the toxicity of aqueous solution during the decomposition processes taking place in swimming pool water installations (Figure 5) due to the formation of, among others, 2,6-di-tert-butylhydroquinone and2,6-di-tert-butylbenzoquinone that can be cytotoxic and genotoxic to diverse cells and animals [51]. 

An interesting phenomenon is the presence of ascorbic acid in swimming pool water. The studies have shown it to be the fourth most frequently consumed of all food additives [52] that, in addition to its antioxidant and nutritional properties, has been investigated as a means for reducing residual halogen-based oxidants [47]. However, the safety for human health of ascorbic acid presence in swimming pool water is questionable and under discussion. Research by the Environmental Protection Agency (EPA) shows that the use of ascorbic acid is one of the effective methods for the dechlorination of water [53]. For this reason, many not fully aware swimming pool users (especially private backyard ones) decide to deliberately introduce this compound into their swimming pools. However, it should be emphasized that such action is not surely beneficial for the quality of pool water and the safety of its use. First of all, chlorine is introduced into the pool to kill bacteria, viruses and microorganisms. Attempting to achieve dechlorination is thus achieving the opposite effect. Moreover, ascorbic acid lowers the pH of the water, which may result in corrosion of the elements, such as ladders, nozzles, skimmers, foil, etc. In addition, it may irritate the eyes and skin of swimmers. It has also been shown that ascorbic acid can be rapidly oxidized to dehydroascorbic acid when added to bicarbonate rich (buffered) copper-contaminated drinking water [54]. Thus, swimmer will most likely ingest dehydroascorbic acid that has been shown to cause oxidative stress and apoptosis in pancreatic and neural cells by depleting their intracellular store of reduced glutathione [55,56,57]. The impact of the long-term intake of dehydroascorbic acid, the oxidized form of ascorbic acid, on human health, still remains to be studied [54].

Sodium benzoate (E211) and Benzoic Acid (E210) are two of the most popular preservatives that can be used in various food products [58]. These are compounds with abroad safety profile and dose-dependent effects that are almost always adverse in the case of high doses [59]. From the use pattern, it can be expected that benzoic acid is released to surface waters, leaching water and groundwater, while no information on the environmental transport and distribution of sodium benzoate could be identified [60]. Owing toits use pattern, which is similar to that of benzoic acid, most of the amounts released to the environment are also expected to be emitted to aquatic compartments (e.g., surface waters). From their physical/chemical properties, they are not expected to volatilize from water and soil to the atmosphere or to adsorb to sediment or soil particles. They both exhibited low to moderate toxicity to aquatic organisms. The lowest reported EC50 value of9 mg/L was determined in a chronic study (14 days) for cell multiplication inhibition by benzoic acid in the cyan bacterium Anabaena inadequacies. EC50/LC50 values for the other aquatic species tested were in the range of 17–1291 mg/L [60]. However, it is believed that benzoates can be transformed by decarboxylation into toxic benzene, especially in combination with ascorbic acid(also detected in tested swimming pools) and then become a compound of high toxicity, mutagenicity and teratogenicity [61]. The hydroxyl radical, formed by the metal-catalysed reduction in O_2_ and H_2_O_2_ by ascorbic acid, can attack benzoic acid to form benzene [62], and the heat and light can increase the rate of benzene formation [63]. It has also been reported that sodium benzoate has a mutagenic and genotoxic effect [64], generates oxidative stress and has an adverse effect on the immune system, liver, kidneys and fertility.

Saccharin, aspartame and sorbitol, due to their environmental persistence and common detection in the environment, have been recognized as compounds of emerging concern [65]. They are some of the most popular artificial sweeteners used in various food products [66], so their presence in the pool water environment seems to be inevitable. Although comprehensive toxicological tests have been conducted and they appear to be nontoxic to humans within regulated concentrations, their unintended presence in the environment still causes considerable concern. They have previously been proven to occur ubiquitously in surface waters, with concentrations ranging from 0.10 mg/L to 0.12 mg/L [67]. They are recognized as a new class of environmental contaminants due to their extreme persistence and ubiquitous nature. The continuous introduction of artificial sweeteners into the aquatic environments is attributed to their resistant behaviour to wastewater treatment processes [68]. However, their behaviour, fate and long-term ecotoxicological contribution in water resources is, by large, still unknown. 

The real impact of aspartame on human health is still unclear. The studies conducted in these field focused mainly on animals [69] and indicate a carcinogenic impact on many species [70]. However, Borghoff et al. [71], after an extensive review of the literature, pointed out that there were no clear or consistent signals of carcinogenicity following aspartame. Bandyopadhyay et al. [72] showed that compounds such as aspartame and sorbitol could induce DNA damage in bone marrow cells. A recent study has also reported that aspartameisis toxic to *Lemna minor, Sinapis alba, Daphnia magna, Enchytraeuscrypticus, Desmodesmussubspicatus* and *Lactuca sativa* (100 mg/kg) and disrupts the reproduction of *Enchytraeidae* [73].

**Figure 6:**
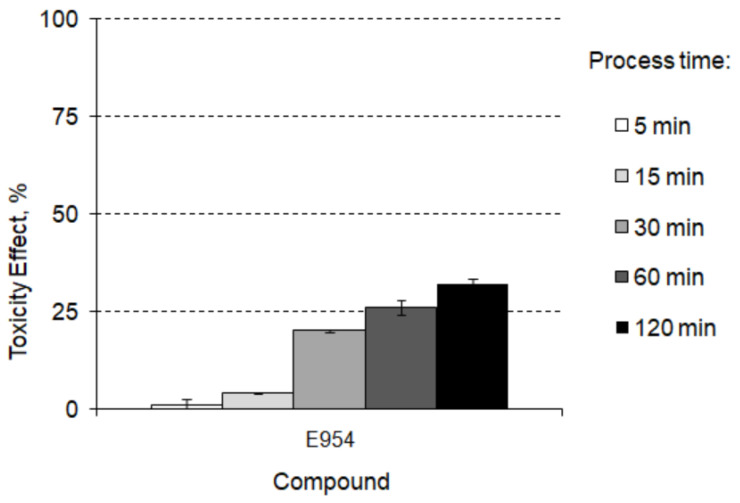
The toxicity change in the saccharine by-product formation pathway in swimming pool water installation.

Luo et al. [74] indicated that saccharincan causes a hazard and risk potential to aquatic organisms, which may also affect human health. Uçar and Yilmaz [75] noted that this type of artificial sweetener may be a weak carcinogen, causing cancer of the urinary tract of male rats. Other studies have proved that saccharin can induce liver inflammation in mice [76] or could be one of the main factors causing paediatric inflammatory bowel disease [77].Studies conducted on the assessment of the possibility of saccharin and other artificial sweeteners decomposition in the chlorination process have shown that most of those compounds were persistent and not transformed by the action of reactive chlorine radicals [78].This may be related toa lack of electron-rich sites for oxidation in the compound molecule [79]. In addition, the action of ultraviolet (UV)irradiation, which is used in the swimming pool water treatment technology as a supporting disinfection process [80], does not allow for a significant increase in the concentration of saccharin in the aquatic environment [81]. On the other hand, Davididou et al. [82] proved the degradation of saccharin under solar radiation. However, this process leads to the production of toxic decomposition by-products with –OH and =O groups. The toxicity change in the saccharine by-product formation pathway in swimming pool water installation is shown in Figure 6.

## 5. Conclusions

The presented study investigated the occurrence of 13 selected micropollutants, classified as Contaminants of Emerging Concern, from the group of food additives in water samples collected from 10 swimming pool systems. The study area was selected based on the lack of available information regarding suspected contamination of swimming pool water by food additives. The variety and concentration of chemical compounds in these aquatic systems can be quite diversified, presenting a challenge in terms of both purification and quality control. This paper provides insights into the concentrations and variability of food additives in various types of swimming pools. The presence of these compounds and the possibility of their accumulation and transformation in swimming pool installations raise questions about the potential threat to the health of swimming pool users.

Thirteen food additives have been identified in the tested pools. They have been classified as colours (Riboflavin, Canthaxanthin), preservatives (Sodium Benzoate, Benzoic Acid), antioxidants (Butylated Hydroxyanisole, Butylated Hydroxytoluene, Tetiary Butylhydroquinone), sweeteners (Saccharin, Aspartame, Sorbitol) and others (Citric Acid, Lactic Acid). No compounds from the group of emulsifiers, stabilizers, thickeners and gelling agents have been identified in this study. In general, food additives were found more often in private than in public swimming pools. All of the food additives identified in the swimming pools were below the LOQs in all of the fill water (fresh water) samples. This implies that contamination of swimming pool water by food ingredients is occurring within the swimming pools themselves.

There are three main ways of exposing swimmer to food additives and their by-products present in swimming pool water (oral route by water swelling, inhalation and direct contact route). The determined hazard factors for swimmers of all ages and genders indicated that the health risk from oral exposure to the food additives present in swimming pools is low, considering exposure to a single contamination with individual compounds. However, it should be highlighted and kept in mind that in swimming pool basins there is co-exposure to countless amounts of different organic micropollutants, which is not taken into account in the implemented health risk assessment methodology. The hazard statements of food additives identified in tested swimming pools indicate that human exposure via different routes may cause not only immediate effects, such as skin or eye irritation and allergic reactions, but also long-lasting effects, e.g., damaged fertility or genetic defects.

## Figures and Tables

**Table 1:** Characteristics of the tested swimming pool facilities.

Parameter	Public Outdoors Swimming Pools					Private Backyards Swimming Pools				
	SP1	SP2	SP3	SP4	SP5	SP6	SP7	SP8	SP9	SP10
Function of Basin	Sport pool	Water Playground	Recreational	Children Pool	Recreational	Recreational	Children Pool	Children Pool	Children Pool	Recreational
Dimensions of the pool basin (m × m)	25 × 12.5	25 × 20	25 × 12.5	25 × 20	11.5 × 10	⌀4.5	⌀5	5 × 10	6 × 3	4 × 2
Depth of the pool basin (m)	1.2	0.1	0.8–1.2	0.1–0.6	1.2	1.2	1.2	0.8–1.3	1.55	1.2
Attendance (person/day)	544	240	476	368	144	1	5	3	3	4
Water temperature (°C)	26	28	30	30	30	29	28	28	27	29
pH	7.0	7.1	7.0	7.1	7.0	6.9	7.2	7.2	7.4	6.8
periodicity of changing of water	1 year (according to requirements)					3 months (summer season)				
input of fresh water	depending on water losses related to splashing and evaporation of water (an average of approx. 30 L/1 user per day)									

**Table 2:** Detailed parameters of the extraction processes.

Superclean^TM^ Extraction Tubes Properties								
Tube type		Envi-8	Envi-18		LC-8	LC-18	LC-CN	LC-Ph
Bed type		C_8_ (octyl)	C_18_ (octadecyl)		C_8_ (octyl)	C_18_ (octadecyl)	Cyano	Phenyl
Bed mass (mg)		1000	1000		500	1000	500	500
Tube volume (mL)		6	6		6	6	6	3
Carbon Loading (%)		14	17		7	11.5	7	5.5
**Solid Phase Extraction Steps**								
Conditioning	solvents	5 mL MeOH	5.0 mL ACN 5.0 mL MeOH	3.0 mL DCM 3.0 mL ACN 3.0 mL MeOH	5.0 mL ACN 5.0 mL MeOH	5.0 mL ACN 5.0 mL MeOH	5.0 mL ACN 5.0 mL MeOH	5.0 mL ACN 5.0 mL MeOH
	velocity	10 mL/min						
Bed washing	matrix	5.0 mL deionized water						
	velocity	10 mL/min						
Sample flow	volume	100 mL						
	velocity	1 mL/min						
Drying		5 min under vacuum						
Elution	solvents	3 mL MeOH	1.5 mL ACN 1.5 mL MeOH	2.0 mL DCM 1.5 mL ACN 1.5 mL MeOH	1.5 mL ACN 1.5 mL MeOH	1.5 mL ACN 1.5 mL MeOH	1.5 mL ACN 1.5 mL MeOH	1.5 mL ACN 1.5 mL MeOH
	velocity	10 mL/min						

**Table 3:** Conditions of chromatographic analysis.

Carrier Gas	Helium 6.0 from SIAD (Ruda Śląska, Poland)	
**Injection velocity**	3 mL/min	
**Injection temperature**	250 °C	325 °C
**Column Type**	SLB^TM^—5 ms	HP—5ms
**Column size**	30 m × 0.25 mm	
**Column film thickness**	0.25 μm	
**Oven temperature** **program**	80 °C held for 6 min	40 °C for 2 min
	5 °C/min up to 260 °C	
	20 °C/min up to 300 °C held for 2 min	10 °C/min up to 300 °C held 10 min
**Transfer line temperature**	250 °C	325 °C
**Ion trap temperature**	150 °C	
**Ion source temperature**	230 °C	
**Ion registration mode**	TIC	
**Ion registration range (m/z)**	50 ÷ 600	

**Table 4:** List of food additives identified in tested swimming pool water samples.

Group	Abbreviation	Compound	CAS	Molar Mass (g/mol)
Antioxidants	E320	Butylated Hydroxyanisole	25013-16-5	180.24
	E321	Butylated Hydroxytoluene	128-37-0	220.35
	E319	TertiaryButylhydroquinone	1948-33-0	166.22
	E300	Ascorbic Acid	50-81-7	176.12
Preservatives	E211	SodiumBenzoate	532-32-1	144.10
	E210	Benzoic Acid	65-85-0	122.12
Sweeteners	E954	Saccharin	81-07-2	183.18
	E951	Aspartame	22839-47-0	294.30
	E420	Sorbitol	50-70-4	182.17
Colours	E101	Riboflavin	83-88-5	376.36
	E161g	Canthaxanthin	514-78-3	564.84
Others	E330	Citric Acid	77-92-9	192.12
	E270	Lactic Acid	50-21-5	90.08

**Table 5:** Validation parameters of the method used for the targeted chromatographic analysis.

Abbreviation	Compound	R ± SD, %	CV	IDL, µg/L	LOQ, µg/L
E320	Butylated Hydroxyanisole	96 ± 3	0.02	0.04 × 10^−3^	3.0
E321	Butylated Hydroxytoluene	99 ± 1	0.01	0.01 × 10^−3^	2.0
E319	Tertiary Butylhydroquinone	97 ± 1	0.01	0.03 × 10^−3^	2.7
E300	Ascorbic Acid	97 ± 2	0.02	0.11 × 10^−3^	15.0
E211	SodiumBenzoate	95 ± 1	0.01	0.01 × 10^−3^	1.8
E210	Benzoic Acid	98 ± 1	0.02	0.36 × 10^−3^	41.2
E954	Saccharin	94 ± 3	0.03	0.24 × 10^−3^	28.2
E951	Aspartame	95 ± 1	0.01	0.18 × 10^−3^	20.4
E420	Sorbitol	95 ± 2	0.01	0.07 × 10^−3^	6.2
E101	Riboflavin	96 ± 2	0.02	0.31 × 10^−3^	36.7
E161g	Canthaxanthin	95 ± 3	0.02	0.22 × 10^−3^	23.3
E330	Citric Acid	97 ± 2	0.03	0.09 × 10^−3^	12.3
E270	Lactic Acid	95 ± 2	0.02	0.04 × 10^−3^	3.0

R—Recovery, CV—Coefficient of Variation, IDL—Instrumental Detection Limit, LOQ—Limit of Quantification.

**Table 7:** Toxicity of the tested food additives to aquatic organisms [38].

**Abbreviation**	**Species Name**	**Parameter**	**Value (mg/L)**	**Test Duration Time (Days)**
E320	*Lepomis macrochirus*	LC50	4.8	2
	*Ictalurus punctatus*	LC50	1.5	2
	*Oryziaslatipes*	LC50	2.5	1
	*Oncorhynchus mykiss*	LC50	1	2
	*Dreissenapolymorpha*	EC50	3.4	2
	*Dreissenapolymorpha*	LC50	65	2
E321	*Daphnia pulex*	EC50	1.44	2
	*Oryziaslatipes*	LC50	5.3	1
	*Tetrahymena pyriformis*	EC50	1.7	1
	*Dreissenapolymorpha*	EC50	1.3	2
	*Daphnia pulex*	EC50	1.44	2
E319	*Ictalurus punctatus*	LC50	0.37	2
	*Lepomis macrochirus*	LC50	0.15	2
	*Oncorhynchus mykiss*	LC50	0.37	2
	*Dreissenapolymorpha*	EC50	1	2
	*Dreissenapolymorpha*	LC50	118	2
E300	*Xenopuslaevis*	EC50	11600	4
	*Xenopuslaevis*	LC50	19200	4
E211	*Asellus intermedius*	LC50	100	4
	*Gammarus fasciatus*	LC50	100	4
	*Daphnia magna*	LC50	100	4
	*Danio rerio*	EC50	68.5	2
	*Danio rerio*	LC50	461	2
	*Pimephalespromelas*	LC50	100	4
	*Girardiatigrina*	LC50	100	4
	*Lumbriculus variegatus*	LC50	100	4
E210	*Raphidocelissubcapitata*	EC50	36.39	2
	*Anabaena variabilis*	EC50	55	0.125
	*Anabaena cylindrica*	EC50	60	0.125
	*Anabaena inaequalis*	EC50	5	0.125
	*Chlorella pyrenoidosa*	EC50	60	0.125
	*Anabaena cylindrica*	EC50	30	0.2083
	*Chlorococcales*	EC50	168	1
	*Raphidocelissubcapitata*	EC50	207.5	2
	*Scenedesmusquadricauda*	EC50	75	0.125
	*Microcystis aeruginosa*	EC50	0.25	3
	*Chlorella vulgaris*	EC50	0.14	3
	*Desmodesmussubspicatus*	EC50	333	7
	*Xenopuslaevis*	EC50	433	4
	*Thamnocephalusplatyurus*	EC50	177	1
	*Daphnia magna*	EC50	100	2
	*Gambusiaaffinis*	LC50	180	4
	*Leuciscusidus ssp. melanotus*	LC50	460	2
	*Pimephalespromelas*	EC50	2809	0.0833
	*Meloidogyne arenaria*	LC50	290.6	1
E954	*Xenopuslaevis*	EC50	0.0141	4
	*Xenopuslaevis*	LC50	0.01303	4
	*Danio rerio*	EC50	4753.6	5.8333
	*Danio rerio*	LC50	7272.3	1.8333
	*Danio rerio*	LC50	5585.2	5.8333
	*Danio rerio*	LC50	7272.3	2.8333
E420	*Lemnagibba*	EC50	27143.9	7
E101	*Raphidocelissubcapitata*	EC50	12	2
E161g	*Leuciscusidus*	LC50	10	4
E330	*Daphnia magna*	EC50	1535	1
	*Carcinusmaenas*	LC50	160	2
	*Leuciscusidus ssp. melanotus*	LC50	440	2
	*Pimephalespromelas*	EC50	2942	0.0833
	*Oncorhynchus mykiss*	EC50	653.2	1
	*Azumiobodohoyamushi*	EC50	100	1
E270	*Moinamicrura*	LC50	329.12	4
	*Oreochromismossambicus*	LC50	257.73	4
	*Meloidogyne arenaria*	LC50	4503.94	1
	*Branchiurasowerbyi*	LC50	50.82	4

## Data Availability

The data presented in this study are available on request from the corresponding author.
